# Supplementary material for: Pre-clinical investigation of astatine-211-parthanatine for high-risk neuroblastoma
Source: Commun Biol. 2022 Nov 17;5:1260. doi: 10.1038/s42003-022-04209-8 (PMC9671962; doi:10.1038/s42003-022-04209-8)
Supplement: Supplementary file 2 — Supplemental Information [file 42003_2022_4209_MOESM2_ESM.pdf]

Supplemental Information

Supplementary Methods

Autoradiography

Injected dose : 10 μCi/500 μL, IP injection

Standard : 0.2 μCi/10 μL (#1) was serial diluted until #10 (#10 was DW only)

Thickness : 20 μm, each section was obtained between 200 μm gap, 3 sections collected

Exposure time : 61 h (from 26, 8:00 pm to 29, 9:00 am)

Compound : [^{211}At]PTT (1 h), [^{211}At]PTT +SSKI (1 h), and free ^{211}At (1 h)

Supplementary Figures

A)

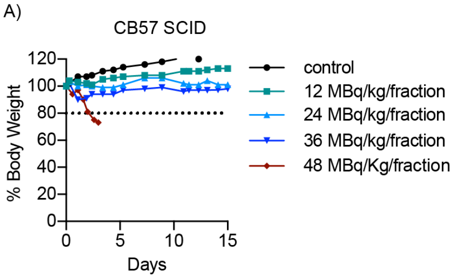

Supplementary Figure 1: Maximum tolerated dose finding studies for [^{211}At]PTT administered as 4 dose fractions given twice weekly in male and female CB57 SCID mice (n=3 per sex per dose level).

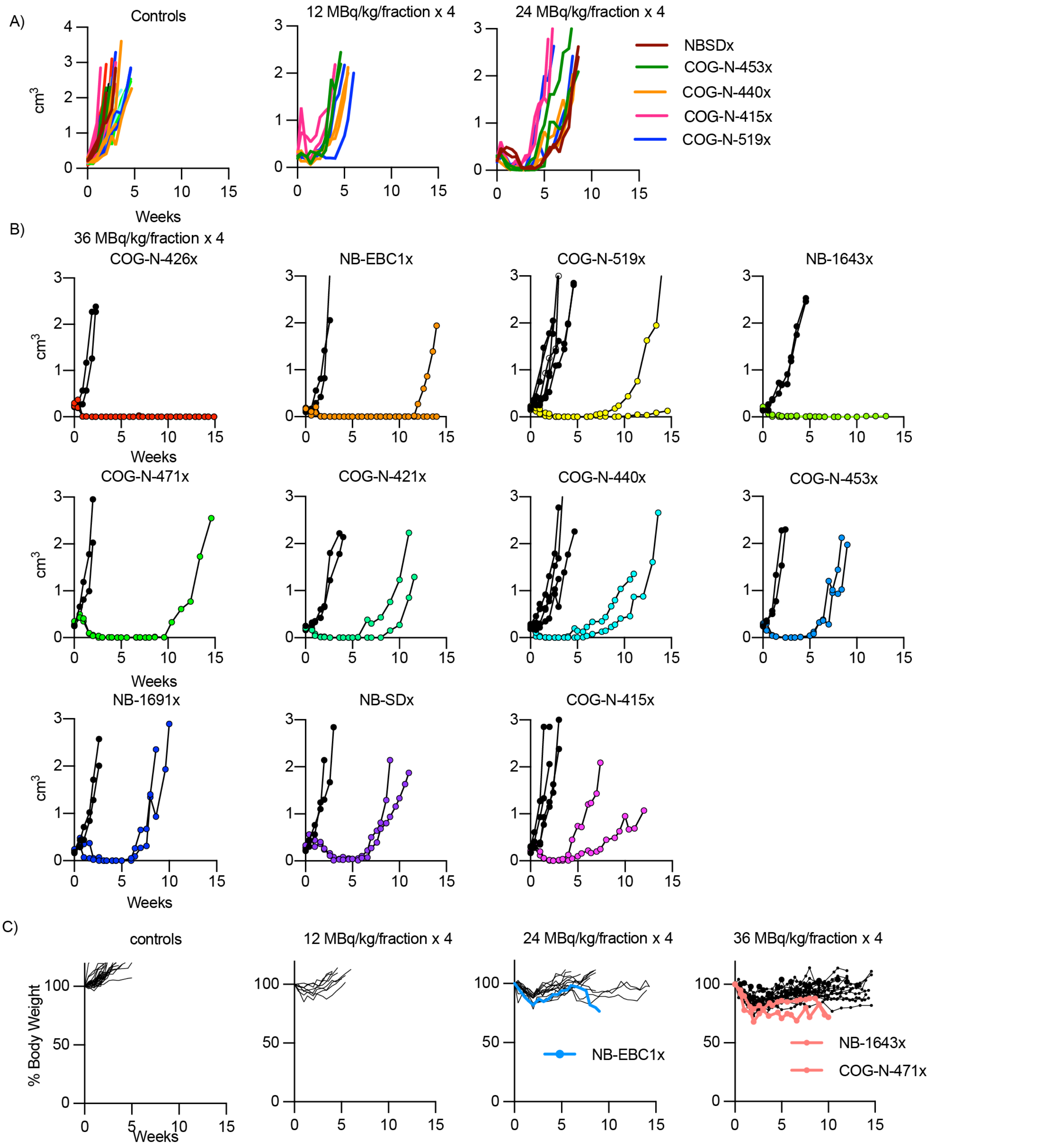

Supplementary Figure 2: a) Spider plots of tumor growth for control and mice treated at 12 MBq/kg/fraction given twice weekly for a total of 4 fractions. **b)** Spider plots for individual models treated at the MTD (black - control; colored lines - 36 MBq/kg/fraction x 4). **c)** Mouse weights shown for control and each treatment group.

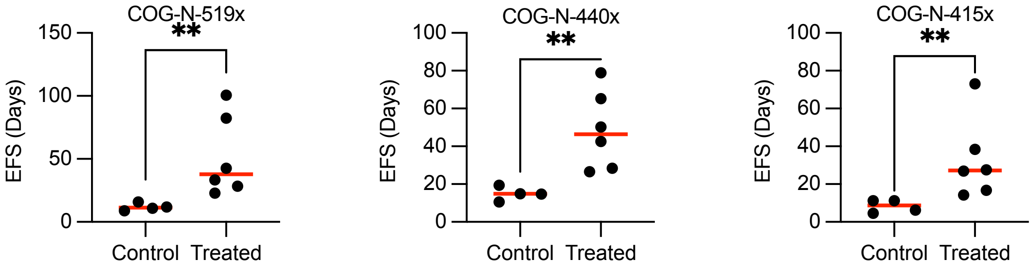

Supplementary Figure 3: Grouped analysis for models treated at all dose levels (COG-n-415x, COG-n-440x, and COG-n-519x; n=6/model dose levels = 12, 24, 36 MBq/kg) vs. control (n=4/model) (T-test, **p-value <0.05).

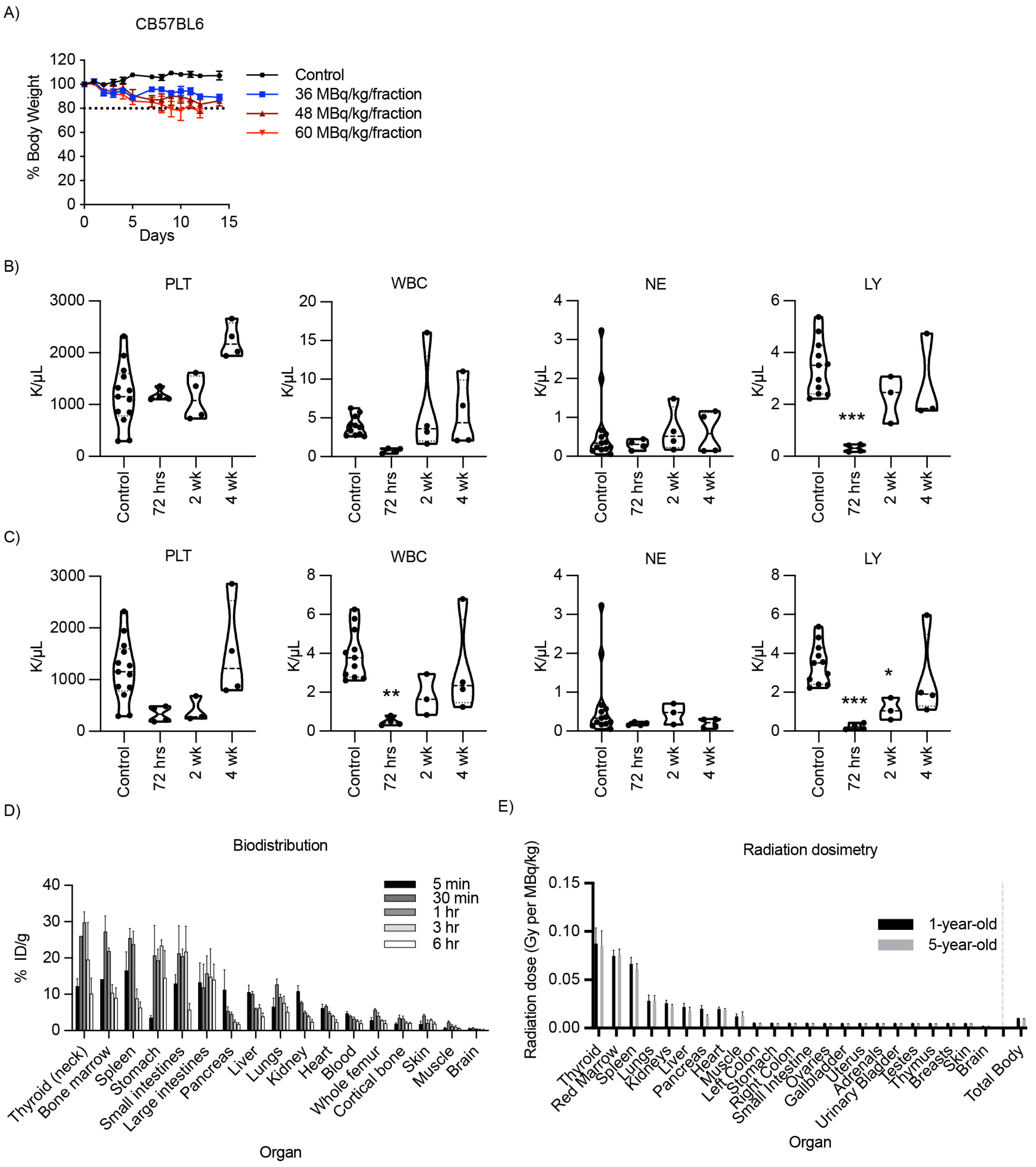

Supplementary Figure 4: Tolerability studies to determine hematological and marrow toxicity of [^{211}At]PTT in CB57BL6 mice. **a)** Maximum tolerated dose finding studies for [^{211}At]PTT administered as 4 dose fractions given twice weekly in CB57BL6 mice. **b)** Complete blood counts assessed at 72

hours, 2 and 4 weeks post-treatment for 2 and **c)** 3 dose fractions of 36 MBq/kg given twice weekly. Statistical analysis was performed by ordinary one-way ANOVA comparison between the mean of control and test groups. p-value denoted as * <0.05, ** <0.01, ***<0.001, ****<0.0001. PLT-platelets, WBC-white blood cells, LY-lymphoctyes, NE-neutrophils. **d)** [^{211}At]PTT Biodistribution data in CB57/BL6 healthy mice with **e)** estimated human dosimetry.

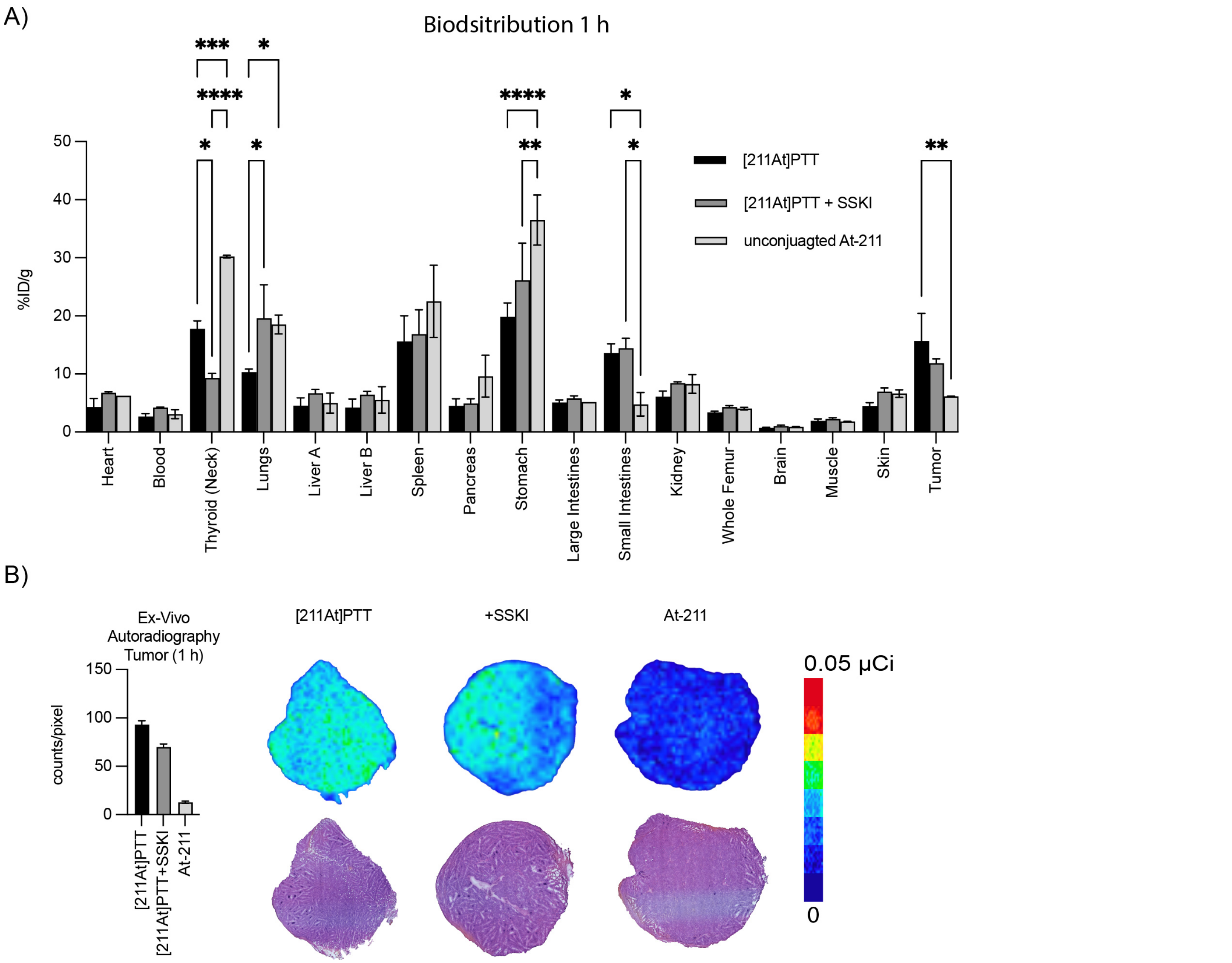

Supplementary Figure 5: a) Biodistribution of [^{211}At]PTT (n=3), [^{211}At]PTT +SSKI (n=2), and unconjugated astatine-211 (n=2) at 1 hour post injection (2-way ANOVA analysis, * denotes significance p-value <0.05). **b)** Ex-vivo autoradiography of NB-EBC1 tumors for each group (n=1 tumor/group and n=3 sections/tumor spaced by 200 µm distance).

	Sex	*Phase*	*Age*	*Inferred Ethnicity*	*Site of Initial Tumor*	*Prior Therapy*
COG-N-415x	Female	Progressing Disease	1.4	European	Adrenal gland	Unknown
COG-N-421x	Male	Relapse	2.73	European	Adrenal gland	Unknown
COG-N-COG-N-426x-Felix	Male	Relapse	4	European	Unknown	Multiple rounds of chemotherapy, radiation, MIBG, immunotherapy; MLN-8237, irinotecan, temozolomide
COG-N-440x	Female	Relapse	0.93	European	Adrenal gland	Unknown
COG-N-453x	Male	Relapse	1.4	European	Adrenal gland	Unknown
COG-N-471x	Female	Relapse	2.58	SouthAsian Or Hispanic	Adrenal gland	Unknown
COG-N-519x	Male	Relapse	2	European	Adrenal gland	Unknown
NB-1643x	Male	Diagnosis	2	European	Retroperitoneal mass	No Prior Tx
NB-1691x	Male	Progressing Disease	Unknown	European	Unknown	AraC, Dauno, 6-TG-VP-16, 5-AzaC
NB-EBc1x	Male	Progressing Disease	2.6	European	Adrenal mass	CTX, DOX, CDDP, VM-26
NB-SDx	Male	Progressing Disease	Unknown	African	Unknown	CTX, DOX, CDDP, VM-26

Supplementary Table 1: Patient demographics for high-risk neuroblastoma patient tumors used for production of patient derived xenograft mouse models (9).

PDX MODEL	MUTATION
COG-N-426X-FELIX	ALK_c.3824G>A_p.R1275Q
NB-EBC1X	KRAS_c.35G>A_p.G12D
*COG-N-519X	TP53_p.G245S
*NB-1643X	ALK_c.3824G>A_p.R1275Q
*COG-N-471X	
*COG-N-421X	
*COG-N-440X	
*NB-SDX	ALK_c.3522C>A_p.F1174L; TP53_c.527G>T_p.C176F
*NB-1691X	PTPN11_c.205G>A_p.E69K
*COG-N-453X	ALK_c.3522C>A:F1174L
*COG-N-415X	ALK_c.3522C>A:F1174L

Supplementary Table 2: Genetic mutations for NB PDX models.

**MYCN* amplified.

PDX MODEL	PARP1 mRNA	EFS							
		Dose (MBq/kg/fraction) N=2							
		36		24		12		Control	
	FPKM	N_1	N_2	N_1	N_2	N_1	N_2	N_1	N_2
**COG-N-426X-FELIX**	52	104.1∞	104.1∞	49.5^{+}59∞	103.9∞			6	11.8
**COG-N-519X**	43	82.4	100.5∞	28.3	42.6	22.7	33.2	10.9	8.8
**COG-N-471X**	93	§64.9∞	83.5					7.7	6.3
**NB-1643X**	139	90.5∞	§76.6∞					16.4	21.5
**NB-SDX**	86	59.4	51.1	44.1	46.5			7.5	10.5
**COG-N-453X**	67	48.6	52.9	43.8	33.4	19.5	22.4	9.4	7.1
**COG-N-440X**	105	78.8	65.1	50.2	42.5	28.3	26.6	10.6	14.9
**NB-EBC1X**	236	90.4	97.6∞	45^{+}47	§58.4			12	9.4
**COG-N-415X**	89	38.3	73	27.6	26.9	14.1	16.	6.3	4.6
**COG-N-421X**	146	61.4	76					13	15.5
**NB-1691X**	202	50.9	78.4					10.1	9.2

Supplementary Table 3: PARP1 mRNA expression and event free survival for PDX models treated with 12, 24, 36 MBq/kg/fraction (maximum tolerated dose) and vehicle control.

∞Tumor free at end of study.

$^{+}$ Re-challenge at 24 MBq/kg/fraction ($EFS^{1} + EFS^{2}$).

§Removed from study for body weight decrease > 20% from start of study.

	1-year-old				5-year-old			
Thyroid	0.078	0.132	0.087	0.053	0.076	0.127	0.084	0.052
Red Marrow	0.073	0.070	0.091	0.065	0.074	0.069	0.093	0.066
Spleen	0.061	0.070	0.084	0.051	0.055	0.063	0.076	0.049
Lungs	0.035	0.025	0.014	0.040	0.034	0.025	0.014	0.039
Kidneys	0.021	0.021	0.034	0.028	0.018	0.018	0.029	0.023
Liver	0.019	0.018	0.018	0.033	0.016	0.015	0.011	0.028
Pancreas	0.023	0.016	0.013	0.028	0.018	0.012	0.009	0.006
Heart	0.017	0.017	0.026	0.020	0.015	0.016	0.024	0.018
Muscle	0.008	0.008	0.016	0.016	0.011	0.012	0.024	0.004
Left Colon	0.005	0.006	0.005	0.005	0.004	0.005	0.005	0.005
Stomach	0.005	0.005	0.005	0.005	0.004	0.005	0.005	0.005
Right Colon	0.005	0.006	0.005	0.005	0.004	0.005	0.005	0.004
Small Intestine	0.005	0.005	0.005	0.005	0.004	0.005	0.005	0.005
Ovaries	0.005	0.005	0.005	0.005	0.004	0.005	0.005	0.004
Gallbladder	0.005	0.005	0.005	0.005	0.004	0.005	0.005	0.004
Uterus	0.005	0.005	0.005	0.005	0.004	0.005	0.005	0.004
Adrenals	0.005	0.005	0.005	0.005	0.004	0.005	0.005	0.004
Urinary Bladder	0.005	0.005	0.005	0.005	0.004	0.005	0.005	0.004
Testes	0.005	0.005	0.005	0.005	0.004	0.005	0.005	0.004
Thymus	0.005	0.005	0.005	0.005	0.004	0.005	0.005	0.004
Breasts	0.005	0.005	0.005	0.005	0.004	0.005	0.005	0.004
Skin	0.005	0.005	0.005	0.005	0.004	0.005	0.005	0.004
Brain	0.002	0.002	0.002	0.002	0.002	0.001	0.002	0.002
Total Body	0.009	0.010	0.010	0.011	0.009	0.009	0.011	0.009

Supplementary Table 4: Estimated human dosimetry from CB57/BL6 mouse biodistribution study. *units = Gy per MBq/kg.

Time point	%Injected Dose/gram		
**0.5**	2.4065	3.551463	6.659899
**1**	8.705292	13.3276	24.85939
**6**	7.985528	8.235966	8.191986
**24**	0.008485	1.144186	1.57829
	**Absorbed Dose (cGy/MBq/kg)**		
**Tumor**	4.5716	5.9806	7.417

Supplementary Table 5: Estimated tumor dosimetry from NB-EBC1 tumor bearing mouse biodistribution study in CB57 SCID mice (n=3/time point).
*units = cGy per MBq/kg.
