# Supplementary material for: Pre-clinical investigation of astatine-211-parthanatine for high-risk neuroblastoma
Source: Commun Biol. 2022 Nov 17;5:1260. doi: 10.1038/s42003-022-04209-8 (PMC9671962; doi:10.1038/s42003-022-04209-8)
Supplement: Supplementary file 3 — Description of Additional Supplementary Files [file 42003_2022_4209_MOESM3_ESM.pdf]

Description of Additional Supplementary Files

File name: Supplementary Data 1
Description: This file contains raw data used to generate figures in the main text of the manuscript.
