# Supplementary material for: Pre-clinical investigation of astatine-211-parthanatine for high-risk neuroblastoma
Source: Commun Biol. 2022 Nov 17;5:1260. doi: 10.1038/s42003-022-04209-8 (PMC9671962; doi:10.1038/s42003-022-04209-8)
Supplement: Supplementary file 5 — Reporting Summary [file 42003_2022_4209_MOESM5_ESM.pdf]

nature portfolio

Corresponding author(s): John M. Maris and Daniel A. Pryma

Last updated by author(s): Aug 20, 2022

Reporting Summary

Nature Portfolio wishes to improve the reproducibility of the work that we publish. This form provides structure for consistency and transparency in reporting. For further information on Nature Portfolio policies, see our Editorial Policies and the Editorial Policy Checklist.

Statistics

For all statistical analyses, confirm that the following items are present in the figure legend, table legend, main text, or Methods section.

n/a	Confirmed	
☐	☒	The exact sample size (*n*) for each experimental group/condition, given as a discrete number and unit of measurement
☐	☒	A statement on whether measurements were taken from distinct samples or whether the same sample was measured repeatedly
☐	☒	The statistical test(s) used AND whether they are one- or two-sided<br>*Only common tests should be described solely by name; describe more complex techniques in the Methods section.*
☐	☒	A description of all covariates tested
☐	☒	A description of any assumptions or corrections, such as tests of normality and adjustment for multiple comparisons
☐	☒	A full description of the statistical parameters including central tendency (e.g. means) or other basic estimates (e.g. regression coefficient) AND variation (e.g. standard deviation) or associated estimates of uncertainty (e.g. confidence intervals)
☐	☒	For null hypothesis testing, the test statistic (e.g. *F*, *t*, *r*) with confidence intervals, effect sizes, degrees of freedom and *P* value noted<br>*Give P values as exact values whenever suitable.*
☒	☐	For Bayesian analysis, information on the choice of priors and Markov chain Monte Carlo settings
☒	☐	For hierarchical and complex designs, identification of the appropriate level for tests and full reporting of outcomes
☒	☐	Estimates of effect sizes (e.g. Cohen's *d*, Pearson's *r*), indicating how they were calculated

Our web collection on statistics for biologists contains articles on many of the points above.

Software and code

Policy information about availability of computer code

Data collection	n/a
Data analysis	n/a

For manuscripts utilizing custom algorithms or software that are central to the research but not yet described in published literature, software must be made available to editors and reviewers. We strongly encourage code deposition in a community repository (e.g. GitHub). See the Nature Portfolio guidelines for submitting code & software for further information.

Data

Policy information about availability of data

All manuscripts must include a data availability statement. This statement should provide the following information, where applicable:

- Accession codes, unique identifiers, or web links for publicly available datasets
- A description of any restrictions on data availability
- For clinical datasets or third party data, please ensure that the statement adheres to our policy

Authors of this manuscript will do their best to accommodate requests for resources or data used or generated. All resources and data will be made available upon request, within reason, to corresponding author Dr. John M. Maris at maris@chop.edu.

Human research participants

Policy information about studies involving human research participants and Sex and Gender in Research.

Reporting on sex and gender	n/a
Population characteristics	n/a
Recruitment	n/a
Ethics oversight	n/a

Note that full information on the approval of the study protocol must also be provided in the manuscript.

Field-specific reporting

Please select the one below that is the best fit for your research. If you are not sure, read the appropriate sections before making your selection.

☒ Life sciences ☐ Behavioural & social sciences ☐ Ecological, evolutionary & environmental sciences

For a reference copy of the document with all sections, see nature.com/documents/nr-reporting-summary-flat.pdf

Life sciences study design

All studies must disclose on these points even when the disclosure is negative.

Sample size	The Maris lab has performed preclinical trials of hundreds of novel therapeutics over the last two decades through the NCI funded Pediatric Preclinical In Vivo Testing Program (PIVOT: https://preclinicalpivot.org/). Through these efforts we have adopted "single mouse testing" to more closely recapitulate a clinical trial (ref: 10.1158/0008-5472.CAN-16-0122). We use this design when we expect a large effect size and have sufficient diversity in xenograft models to explore hypotheses such as biomarkers of response or resistance. We often modify to a N=2 design if the mechanisms of synthesis or delivery are complicated (ref: 10.1158/1078-0432.CCR-20-4221). Single mouse or N=2 are now commonly deployed through the PIVOT and Maris lab preclinical testing programs.
Data exclusions	Mice that experienced toxicity defined by weight loss >20% were removed from data analysis.
Replication	n/a
Randomization	Tumor bearing mice were randomized to treatment or control at the time of study enrollment.
Blinding	n/a

Reporting for specific materials, systems and methods

We require information from authors about some types of materials, experimental systems and methods used in many studies. Here, indicate whether each material, system or method listed is relevant to your study. If you are not sure if a list item applies to your research, read the appropriate section before selecting a response.

Materials & experimental systems

n/a	Involved in the study	
☒	☐	Antibodies
☒	☐	Eukaryotic cell lines
☒	☐	Palaeontology and archaeology
☐	☒	Animals and other organisms
☒	☐	Clinical data
☒	☐	Dual use research of concern

Methods

n/a	Involved in the study	
☒	☐	ChIP-seq
☒	☐	Flow cytometry
☒	☐	MRI-based neuroimaging

Animals and other research organisms

Policy information about studies involving animals; ARRIVE guidelines recommended for reporting animal research, and Sex and Gender in Research

Laboratory animals	SCID CB17 and CB57/BL6 mice were used in this study
Wild animals	none
Reporting on sex	we report sex of mice used for each specific experiment. Healthy female and male CB57/BL6 mice were used for toxicity analysis. Female SCID CB17 mice were used for efficacy studies.
Field-collected samples	none
Ethics oversight	All animal studies were approved by the Children's Hospital of Philadelphia Institutional Animal Care and Use Committee. Animal experiments were conducted in an accredited pathogen-free facility at the Department of Veterinary Research (DVR), Children's Hospital of Philadelphia. PDX models were all characterized with DNA and RNA sequencing (17)

Note that full information on the approval of the study protocol must also be provided in the manuscript.
